# DFT and CV data of 4-phenyl-substituted dichloro(bis{2-[1-(phenyl)-1H-1,2,3-triazol-4-yl-κN^3^]pyridine-κN})iron(II) coordination compounds

**DOI:** 10.1016/j.dib.2018.10.085

**Published:** 2018-10-27

**Authors:** J. Conradie, M.M. Conradie, Z. Mtshali, J.H. Potgieter

**Affiliations:** a Department of Chemistry, University of the Free State, P.O. Box 339, Bloemfontein 9300, South Africa; b Division of Chemistry and Environmental Science, Manchester Metropolitan University, Manchester M1 5GD, UK; c School of Chemical and Metallurgical Engineering, University of the Witwatersrand, Private Bag X3, Wits 2050, South Africa

## Abstract

The data presented in this paper are related to the research article entitled “Synthesis, characterisation and electrochemistry of eight Fe coordination compounds containing substituted 2-(1-(4-R-phenyl-1H-1,2,3-triazol-4-yl)pyridine ligands, R=CH_3_, OCH_3_, COOH, F, Cl, CN, H and CF_3_.” (Conradie et al., 2019) [1]. This paper presents electrochemical and density functional theory data of 4-phenyl-substituted dichloro(bis{2-[1-(4-R-phenyl)-1H-1,2,3-triazol-4-yl-κN^3^]pyridine-κN})iron(II) compounds, containing differently substituted 2-(1-(4-R-phenyl)-1H-1,2,3-triazol-1-yl)pyridine ligands (L^2^ – L^9^) (Tawfiq et al., 2014) [2]. Density functional theory calculated data of five different structural isomers for each compound, consistently show that the title compounds are octahedral and that the isomer with the chloride atoms, the pyridine nitrogens and the triazol nitrogens trans to each other, has the lowest energy. Natural bonding orbital (NBO) data and quantum theory of atoms in molecules (QTAIM) data of dichloro(bis{2-[1-(phenyl)-1H-1,2,3-triazol-4-yl-κN^3^]pyridine-κN})iron(II) show origin for the preference of the *trans* isomer.

**Specifications table**

**Table:** 

Subject area	*Chemistry*
More specific subject area	*Computational chemistry and electrochemistry*
Type of data	*Table, text file, graph, figure*
How data was acquired	*BAS 100B/W electrochemical analyzer (Electrochemical studies) and Gaussian 09 (DFT calculations).*
Data format	*Raw and Analyzed.*
Experimental factors	*Samples was used as synthesized. The solvent-electrolyte solution in the electrochemical cell was degassed with Ar for 10 minutes, the sample was added, the sample-solvent-electrolyte solution was then degassed for another 2 minutes and the cell was kept under a blanket of purified argon during the electrochemical experiments.*
Experimental features	*All electrochemical experiments were done in a 2 ml electrochemical cell containing three-electrodes (a glassy carbon working electrode, a Pt auxiliary electrode and a Ag/Ag*^*+*^*reference electrode), connected to a BAS 100B/W electrochemical analyzer. Data obtained were exported to excel for analysis and diagram preparation. DFT data was obtained with the Gaussian 09 program on the High Performance Computing facility of the University of the Free State.*
Data source location	*Department of Chemistry, University of the Free State, Nelson Mandela Street, Bloemfontein, South Africa.*
Data accessibility	*Data is with article.*
	*J. Conradie, M.M. Conradie, Z. Mtshali, D. van der Westhuizen, K.M. Tawfiq, M.J. Al-Jeboori, S.J. Coles, C. Wilson and J.H. Potgieter, Synthesis, characterisation and electrochemistry of eight Fe coordination compounds containing substituted 2-(1-(4-R-phenyl-1H-1,2,3-triazol-4-yl)pyridine ligands, R=CH*_*3*_*, OCH*_*3*_*, COOH, F, Cl, CN, H and CF*_*3*_*., Inorganica Chimica Acta, 484 (2019) 375–385.*

**Value of the data**

• This data provide DFT optimized structures, properties and energies for the different structural isomers of eight differently R-substituted dichloro(bis{2-[1-(4-R-phenyl)-1H-1,2,3-triazol-4-yl-κN^3^]pyridine-κN})iron(II) coordination compounds.
• Natural bonding orbital data of dichloro(bis{2-[1-(phenyl)-1H-1,2,3-triazol-4-yl-κN^3^]pyridine-κN})iron(II) show origin for the preference of the trans isomer.
• Quantum theory of atoms in molecules data of dichloro(bis{2-[1-(phenyl)-1H-1,2,3-triazol-4-yl-κN^3^]pyridine-κN})iron(II) show origin for the preference of the trans isomer.
• The 4-phenyl-R-substituent in dichloro(bis{2-[1-(4-R-phenyl)-1H-1,2,3-triazol-4-yl-κN^3^]pyridine-κN})iron(II) does not show an observable influence on the values of the data.
• This data provide cyclic voltammograms at different scan rates of the Fe^II/III^ redox couple of eight differently R-substituted dichloro(bis{2-[1-(4-R-phenyl)-1H-1,2,3-triazol-4-yl-κN^3^]pyridine-κN})iron(II) coordination compounds.

## 1 Data

**Scheme 1:**
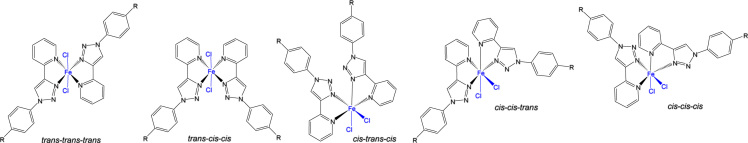
Structure and isomers of Dichloro(bis{2-[1-(4-R-phenyl)-1H-1,2,3-triazol-4-yl-κN^3^]pyridine-κN})iron(II) coordination compounds [Fe(L^n^)_2_Cl_2_] with R=CH_3_ (L^2^), OCH_3_ (L^3^), COOH (L^4^), F (L^5^), Cl (L^6^), CN (L^7^), H (L^8^) and CF_3_ (L^9^) [2]. The isomers are defined by the relative positions of (i) Cl, (ii) N_pyridyl_ and (iii) N_triazole_ around Fe.

The dichloro(bis{2-[1-(4-R-phenyl)-1H-1,2,3-triazol-4-yl-κN^3^]pyridine-κN})iron(II) coordination compounds, [Fe(L^n^)_2_Cl_2_], of this report contain different substituents R on the *para* position of the phenyl groups of the compounds, namely R=CH_3_ (L^2^), OCH_3_ (L^3^), COOH (L^4^), F (L^5^), Cl (L^6^), CN (L^7^), H (L^8^) and CF_3_ (L^9^), see Scheme 1. Five structural isomers are possible for each of the [Fe(L^n^)_2_Cl_2_] compounds, depending on the mutual orientation of the two chloride atoms and the two bidentate (1,2,3-triazol-4-yl)pyridine ligands to each other.

**Fig. 1:**
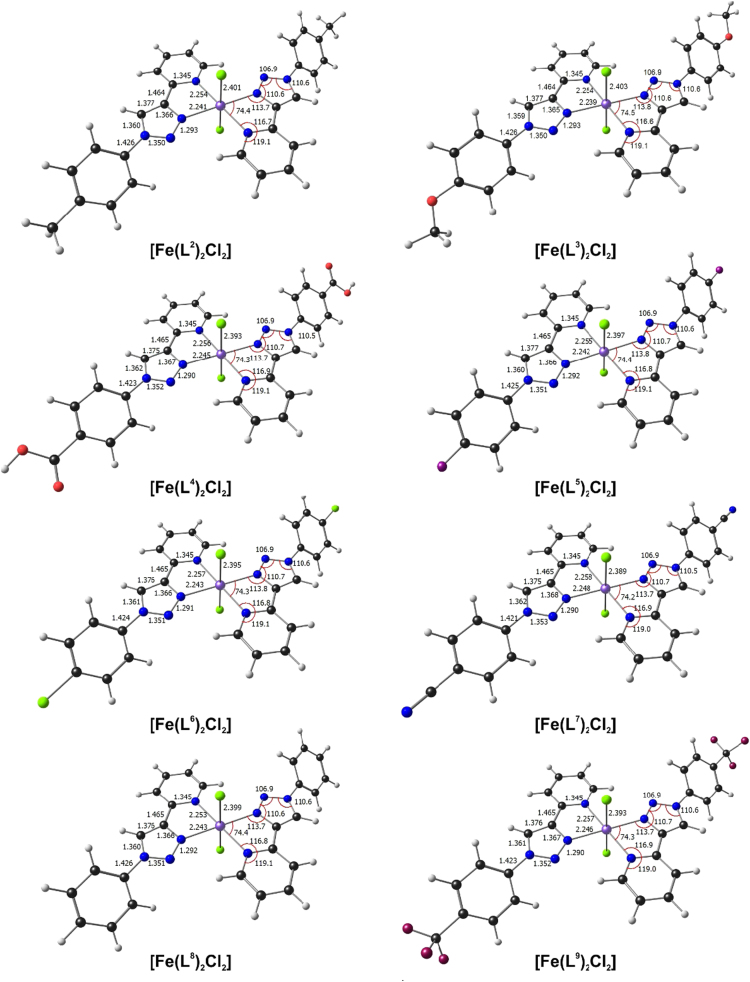
B3LYP calculated optimized geometries of the lowest energy *trans-trans-trans* isomer of [Fe^II^(L^n^)_2_Cl_2_]. R = CH_3_ (L^2^) [3], OCH_3_ (L^3^), COOH (L^4^), F (L^5^), Cl (L^6^), CN (L^7^), H (L^8^) [1] and CF_3_ (L^9^). Colour code of atoms (online version): Fe (purple), Cl (green), F (maroon), O (red), N (blue), C (black), H (white).

The computational chemistry calculated relative energies, obtained for the different structural isomers of each compound, are given in Table 1. For each compound, the *trans-trans-trans* isomer has the lowest energy. This is in agreement with experimental structural data for [Fe(L^3^)_2_Cl_2_] where R=OCH_3_ [1]. The energies of the different isomers, relative to the lowest energy *trans-trans-trans* isomer, are very similar, suggesting that the different R substituent does not have a big influence on the relative stability of the isomers. The optimized structure of the lowest energy *trans-trans-trans* isomer of each compound, with selected structural data is shown in Fig. 1.

**Table 1:** Relative B3LYP/6–311G(d,p) calculated electronic energy ΔE (eV) for the indicated geometrical isomers of high spin (S = 2) [Fe^II^(L^n^)_2_Cl_2_]. R=CH_3_ (L^2^), OCH_3_ (L^3^), COOH (L^4^), F (L^5^), Cl (L^6^), CN (L^7^), H (L^8^) and CF_3_ (L^9^). The energy of the lowest energy isomer is indicated in bolt font.

Isomer	Relative Energy (eV)							
	L^2^^a^	L^3^	L^4^	L^5^	L^6^	L^7^	L^8^^a^^,^^b^	L^9^
*ctc*	0.48	0.46	0.45	0.47	0.47	0.46	0.48	0.47
*ccc*	0.27	0.27	0.26	0.27	0.27	0.26	0.27	0.26
*cct*	0.10	0.11	0.09	0.11	0.11	0.10	0.10	0.10
*ttt*	**0.00**	**0.00**	**0.00**	**0.00**	**0.00**	**0.00**	**0.00**	**0.00**
*tcc*	0.20	0.21	0.17	0.19	0.18	0.17	0.19	0.18

a Data from Ref. [3].

b Data from Ref. [1].

**Fig. 2:**
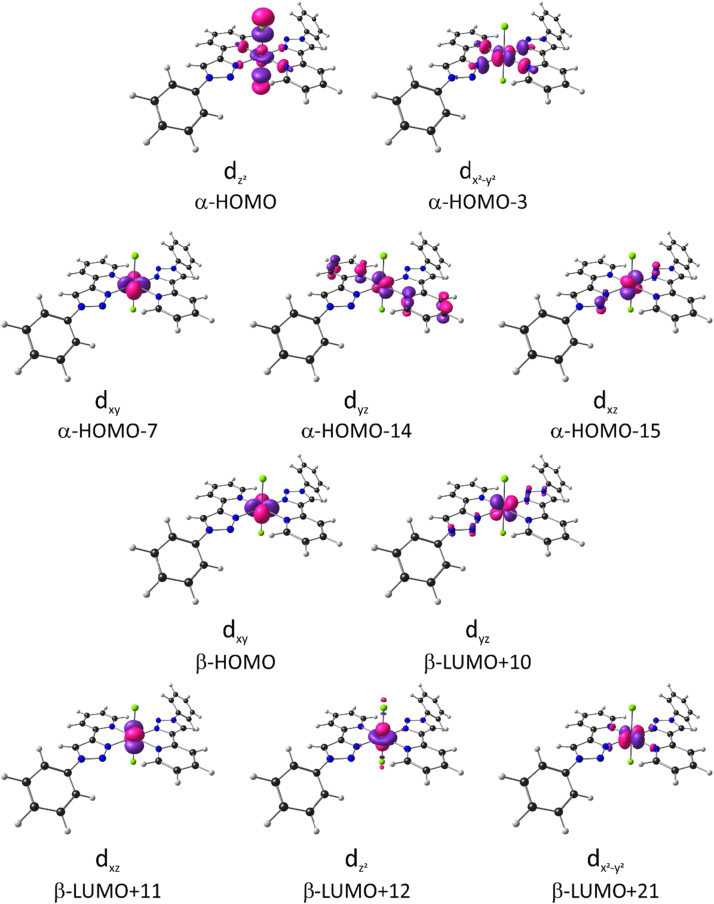
B3LYP calculated Fe-d-based molecular orbitals of the lowest energy *trans-trans-trans* isomer of dichloro{bis[2-(1-phenyl-1H-1,2,3-triazol-4-yl-κN^3^]pyridine-κN]}iron(II), [Fe^II^(L^8^)_2_Cl_2_]. Colour code of atoms (online version): Fe (purple), Cl (green), N (blue), C (black), H (white).

**Fig. 3:**
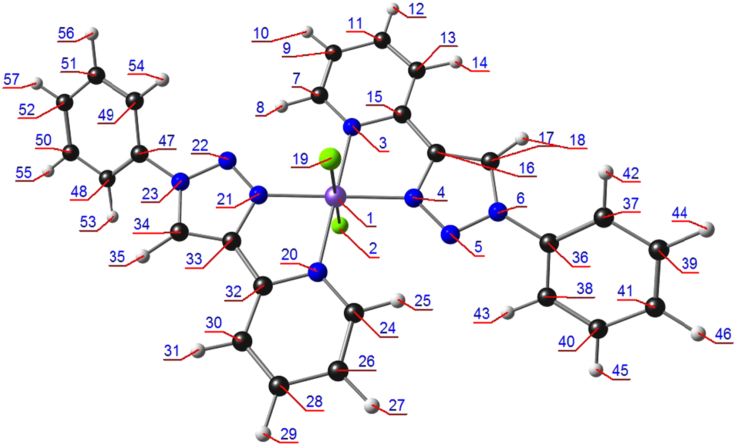
Atom numbering of dichloro{bis[2-(1-phenyl-1H-1,2,3-triazol-4-yl-κN^3^]pyridine-κN]}iron(II), [Fe^II^(L^8^)_2_Cl_2_] used in Tables 2, 3, and 5. Colour code of atoms (online version): Fe (purple), Cl (green), N (blue), C (black), H (white).

The Fe(II) complexes of this study are high spin S = 2 [1], with occupation $d_{\mathrm{xy}}^{2}d_{\mathrm{xz}}^{1}d_{\mathrm{yz}}^{1}d_{z^{2}}^{1}d_{{x^{2}-y}^{2}}^{1}$. The 6 occupied and 4 unoccupied molecular orbitals of mainly Fe-d character of dichloro{bis[2-(1-phenyl-1H-1,2,3-triazol-4-yl-κN^3^]pyridine-κN]}iron(II), [Fe^II^(L^*8*^)_2_Cl_2_] (containing L^8^ with R = H), are shown in Fig. 2. The orbital energy data for the frontier orbitals including the molecular orbitals of mainly Fe-d character are given in Table 2 (Atom numbering used in Table 2 is indicated in Fig. 3). The data for the atomic contributions to the molecular orbitals are also given. The Mulliken charges and spin densities data of non-hydrogen atoms are given in Table 3 (Atom numbering used in Table 3 is indicated in Fig. 3). The spin of 3.735 e^-^ on Fe is consistent with a high spin Fe(II) center containing four unpaired electrons, in [Fe^II^(L^8^)_2_Cl_2_] ).

**Table 2:** Energy (in a.u.) of and atomic contributions to selected frontier molecular orbitals of Dichloro{bis[2-(1-phenyl-1H-1,2,3-triazol-4-yl-κN^3^]pyridine-κN]}iron(II), [Fe^II^(L^8^)_2_Cl_2_] (containing L^8^ with R = H). Atom numbering is indicated in Fig. 3.

	Alpha molecular orbitals		Beta molecular orbitals	
	energy	atomic contributions	energy	atomic contributions
HOMO-15	−0.301	Fe1-d=0.65	−0.304	N3-p=0.14 N20-p=0.14 C11-p=0.12 C28-p=0.12 C13-p=0.11 C30-p=0.11
HOMO-14	−0.289	Fe1-d=0.46	−0.303	N3-p=0.12 N20-p=0.12
HOMO-13	−0.283	C37-p=0.13 C48-p=0.13 C40-p=0.13 C51-p=0.13 C38-p=0.12 C49-p=0.12 C39-p=0.11 C50-p=0.11	−0.302	N3-p=0.23 N20-p=0.23
HOMO-12	−0.283	C48-p=0.13 C37-p=0.13 C51-p=0.13 C40-p=0.13 C49-p=0.12 C38-p=0.12 C50-p=0.11 C39-p=0.11	−0.283	C37-p=0.13 C48-p=0.13 C40-p=0.13 C51-p=0.13 C38-p=0.12 C49-p=0.12 C39-p=0.11 C50-p=0.11
HOMO-11	−0.275	C41-p=0.11 C52-p=0.11 C36-p=0.10 C47-p=0.10	−0.283	C48-p=0.13 C37-p=0.13 C51-p=0.13 C40-p=0.13 C49-p=0.12 C38-p=0.12 C50-p=0.11 C39-p=0.11
HOMO-10	−0.274	C52-p=0.10	−0.276	C41-p=0.11 C52-p=0.11 C36-p=0.10 C47-p=0.10
HOMO-9	−0.250	C26-p=0.08	−0.275	C52-p=0.11 C41-p=0.11 C47-p=0.10 C36-p=0.10
HOMO-8	−0.250	C9-p=0.08	−0.254	Cl19-p=0.37 Cl2-p=0.37 Fe1-d=0.14
HOMO-7	−0.244	Fe1-d=0.95	−0.251	C26-p=0.08
HOMO-6	−0.238	Cl2-p=0.46 Cl19-p=0.46	−0.25	C9-p=0.08
HOMO-5	−0.212	Cl19-p=0.49 Cl2-p=0.49	−0.23	Cl2-p=0.47 Cl19-p=0.47
HOMO-4	−0.212	Cl19-p=0.48 Cl2-p=0.48	−0.213	Cl19-p=0.46 Cl2-p=0.46
HOMO-3	−0.209	Fe1-d=0.58	−0.213	Cl19-p=0.47 Cl2-p=0.47
HOMO-2	−0.206	Cl2-p=0.46 Cl19-p=0.46	−0.208	Cl2-p=0.49 Cl19-p=0.49
HOMO-1	−0.205	Cl2-p=0.46 Cl19-p=0.46	−0.207	Cl2-p=0.48 Cl19-p=0.48
HOMO	−0.182	Fe1-d=0.29 Cl2-p=0.27 Cl19-p=0.27	−0.163	Fe1-d=0.94
LUMO	−0.062	C17-p=0.10 C34-p=0.10	−0.062	C17-p=0.09
LUMO+1	−0.061	C34-p=0.10 C17-p=0.10	−0.061	C34-p=0.10 C17-p=0.10
LUMO+2	−0.054	C28-p=0.08	−0.057	N22-p=0.08
LUMO+3	−0.051	C11-p=0.07	−0.053	C11-p=0.08
LUMO+4	−0.036	C39-p=0.13 C50-p=0.13 C38-p=0.12 C49-p=0.12 C40-p=0.11 C51-p=0.11	−0.036	C39-p=0.09
LUMO+5	−0.036	C50-p=0.13 C39-p=0.13 C49-p=0.12 C38-p=0.12 C51-p=0.10 C40-p=0.10	−0.036	C50-p=0.13 C39-p=0.13 C49-p=0.12 C38-p=0.12 C51-p=0.11 C40-p=0.11
LUMO+6	−0.035	C30-p=0.13 C13-p=0.13 C24-p=0.13 C7-p=0.13	−0.035	C30-p=0.08
LUMO+7	−0.034	C13-p=0.14 C30-p=0.14 C7-p=0.14 C24-p=0.14	−0.034	C13-p=0.13 C30-p=0.13 C7-p=0.13 C24-p=0.13
LUMO+8	−0.006	N22-p=0.09	−0.011	Fe1-d=0.18
LUMO+9	−0.006	N5-p=0.09	−0.006	N22-p=0.09
LUMO+10	0.025	H35-s=0.17 H18-s=0.17 H53-s=0.12 H42-s=0.12 H31-s=0.10 H14-s=0.10	0.011	Fe1-d=0.71
LUMO+11	0.026	H18-s=0.16 H35-s=0.16 H42-s=0.15 H53-s=0.15	0.014	Fe1-d=0.80
LUMO+12	0.036	H31-s=0.15 H14-s=0.15	0.021	Fe1-d=0.65
LUMO+13	0.036	H14-s=0.12 H31-s=0.12	0.025	Fe1-d=0.15 H35-s=0.12 H18-s=0.12 H53-s=0.10 H42-s=0.10
LUMO+14	0.050	C33-p=0.08	0.026	H18-s=0.16 H35-s=0.16 H42-s=0.15 H53-s=0.15
LUMO+15	0.051	C16-p=0.08	0.036	H31-s=0.15 H14-s=0.15
LUMO+16	0.052	H12-s=0.10 H29-s=0.10	0.037	H14-s=0.12 H31-s=0.12
LUMO+17	0.053	H29-s=0.10 H12-s=0.10	0.05	H35-s=0.13 H18-s=0.13 Fe1-d=0.11 H27-s=0.10 H10-s=0.10
LUMO+18	0.065	H44-s=0.15 H55-s=0.15 H45-s=0.14 H56-s=0.14 H43-s=0.11 H54-s=0.11	0.052	H45-s=0.07
LUMO+19	0.069	H55-s=0.20 H44-s=0.20 H56-s=0.12 H45-s=0.12 H54-s=0.12 H43-s=0.12	0.052	H29-s=0.08
LUMO+20	0.073	H46-s=0.11 H57-s=0.11 H10-s=0.11 H27-s=0.11 H14-s=0.11 H31-s=0.11	0.056	C33-p=0.12 C16-p=0.12
LUMO+21	0.083	H57-s=0.21 H46-s=0.21 H54-s=0.12 H43-s=0.12 H53-s=0.11 H42-s=0.11	0.064	Fe1-d=0.70

**Table 3:** Mulliken charges and spin densities of non-hydrogen atoms in dichloro{bis[2-(1-phenyl-1H-1,2,3-triazol-4-yl-κN^3^]pyridine-κN]}iron(II), [Fe^II^(L^8^)_2_Cl_2_] (containing L^8^ with R = H). Atom numbering is indicated in Fig. 3.

Atom No.		charge	spin	Atom No.		charge	spin
1	Fe	1.385	3.735	26	C	−0.188	0.001
2	Cl	−0.767	0.088	28	C	0.005	−0.001
3	N	−0.531	0.019	30	C	−0.098	0.001
4	N	−0.380	0.014	32	C	0.248	0.003
5	N	0.001	0.001	33	C	0.118	0.001
6	N	−0.336	0.003	34	C	0.141	0.001
7	C	0.164	-0.003	36	C	0.184	0.001
9	C	−0.188	0.001	37	C	−0.084	0.000
11	C	0.005	-0.001	38	C	−0.042	0.000
13	C	−0.098	0.001	39	C	−0.097	0.000
15	C	0.248	0.003	40	C	−0.097	0.000
16	C	0.118	0.001	41	C	−0.078	0.000
17	C	0.141	0.001	47	C	0.184	0.001
19	Cl	−0.767	0.088	48	C	−0.084	0.000
20	N	−0.531	0.019	49	C	−0.042	0.000
21	N	−0.380	0.014	50	C	−0.097	0.000
22	N	0.001	0.001	51	C	−0.097	0.000
23	N	−0.336	0.003	52	C	−0.078	0.000
24	C	0.164	−0.003

**Fig. 4:**
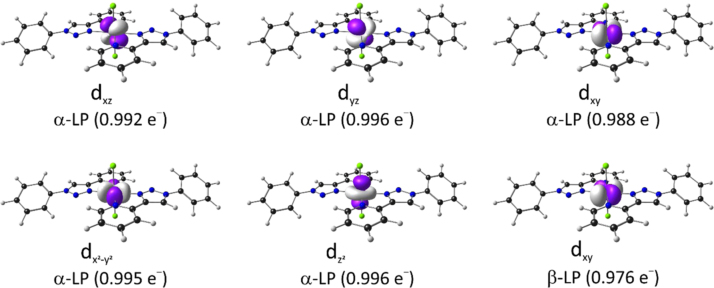
B3LYP optimized geometry of dichloro{bis[2-(1-phenyl-1H-1,2,3-triazol-4-yl-κN^3^]pyridine-κN]}iron(II), [Fe^II^(L^8^)_2_Cl_2_], showing the LP NBOs on Fe, including the NBO electron occupation of the indicated NBOs. Colour code of atoms (online version): Fe (purple), Cl (green), N (blue), C (black), H (white).

**Fig. 5:**
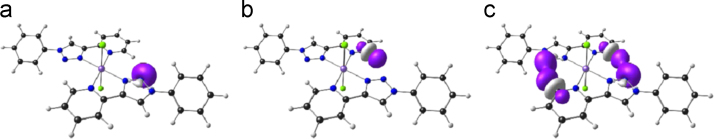
B3LYP optimized geometry of dichloro{bis[2-(1-phenyl-1H-1,2,3-triazol-4-yl-κN^3^]pyridine-κN]}iron(II), [Fe^II^(L^8^)_2_Cl_2_], showing (a) a LP(N) NBO, (b) a BD*(C-H) NBO and (c) the two LP(N) -> BD*(C-H) natural bonding orbital interactions of 0.75 kJ/mol each. Colour code of atoms (online version): Fe (purple), Cl (green), N (blue), C (black), H (white).

Natural bonding orbital (NBO) data for Fe and the atoms bonded to Fe in dichloro{bis[2-(1-phenyl-1H-1,2,3-triazol-4-yl-κN^3^]pyridine-κN]}iron(II), [Fe^II^(L^*8*^)_2_Cl_2_] (containing L^8^ with R=H), are given in Tables 4 and 5 (Atom numbering used in Tables 4 and 5 is indicated in Fig. 3). The six LP NBOs of Fe is shown in Fig. 4. The NBO data show that the lowest energy isomer, the *trans-trans-trans* isomer is stabilized by interaction between a triazole N and a nearby pyridine H, see Fig. 5 for an illustration of the interaction between the lone pair on the triazole N, LP(N), and the antibonding orbital of the nearby pyridine (C-H), BD*(C-H).

**Table 4:** Summary of natural population analysis of selected atoms for dichloro{bis[2-(1-phenyl-1H-1,2,3-triazol-4-yl-κN^3^]pyridine-κN]}iron(II), [Fe^II^(L^8^)_2_Cl_2_] (containing L^8^ with R = H). Atom numbering is indicated in Fig. 3.

Atom	No	Natural Charge	Core	Valence	Rydberg	Total
total values						
Fe	1	1.479	17.997	6.514	0.010	24.521
Cl	2	−0.807	10.000	7.802	0.005	17.807
N	3	−0.524	1.999	5.502	0.023	7.524
N	4	−0.314	1.999	5.281	0.033	7.314
Cl	19	−0.807	10.000	7.802	0.005	17.807
N	20	−0.524	1.999	5.502	0.023	7.524
N	21	−0.314	1.999	5.281	0.033	7.314

alpha spin orbitals						
Fe	1	−1.122	8.998	5.119	0.005	14.122
Cl	2	−0.447	5.000	3.945	0.002	8.947
N	3	−0.275	1.000	2.764	0.011	3.775
N	4	−0.168	1.000	2.652	0.016	3.668
Cl	19	−0.447	5.000	3.945	0.002	8.947
N	20	−0.275	1.000	2.764	0.011	3.775
N	21	−0.168	1.000	2.652	0.016	3.668

beta spin orbitals						
Fe	1	2.601	8.998	1.396	0.005	10.399
Cl	2	−0.360	5.000	3.857	0.002	8.860
N	3	−0.249	1.000	2.738	0.011	3.749
N	4	−0.146	1.000	2.630	0.017	3.646
Cl	19	−0.360	5.000	3.857	0.002	8.860
N	20	−0.249	1.000	2.738	0.011	3.749
N	21	−0.146	1.000	2.630	0.017	3.646

**Table 5:** Natural electron configuration of selected atoms for dichloro{bis[2-(1-phenyl-1H-1,2,3-triazol-4-yl-κN^3^]pyridine-κN]}iron(II), [Fe^II^(L^8^)_2_Cl_2_] (containing L^8^ with R=H). Atom numbering is indicated in Fig. 3.

Atom	No	Natural Electron Configuration
total values		
Fe	1	[core]4S( 0.29)3d( 6.21)4p(0.01)4d( 0.01)
Cl	2	[core]3S(1.97)3p(5.83)
N	3	[core]2S(1.35)2p(4.15)3p(0.02)
N	4	[core]2S(1.37)2p(3.91)3p( 0.02)3d(0.01)
Cl	19	[core]3S(1.97)3p(5.83)
N	20	[core]2S(1.35)2p(4.15)3p(0.02)
N	21	[core]2S(1.37)2p(3.91)3p( 0.02)3d(0.01)

alpha spin orbitals		
Fe	1	[core]4S(0.15)3d(4.96)4p(0.01)
Cl	2	[core]3S(0.99)3p(2.96)
N	3	[core]2S(0.68)2p( 2.09)3p(0.01)
N	4	[core]2S(0.69)2p(1.96)3p(0.01)
Cl	19	[core]3S(0.99)3p(2.96)
N	20	[core]2S(0.68)2p(2.09)3p(0.01)
N	21	[core]2S(0.69)2p(1.96)3p(0.01)

beta spin orbitals		
Fe	1	[core]4S(0.14)3d(1.25)4p(0.01)
Cl	2	[core]3S( 0.98)3p(2.87)
N	3	[core]2S( 0.67)2p(2.07)3p( 0.01)
N	4	[core]2S( 0.68)2p(1.95)3p( 0.01)
Cl	19	[core]3S( 0.98)3p(2.87)
N	20	[core]2S( 0.67)2p(2.07)3p( 0.01)
N	21	[core]2S( 0.68)2p(1.95)3p( 0.01)

**Fig. 6:**
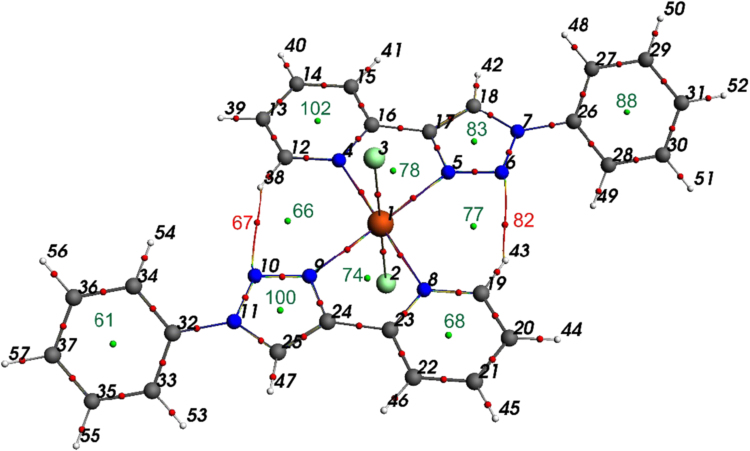
B3LYP optimized geometry of [Fe^II^(L^8^)_2_Cl_2_], showing the QTAIM determined intramolecular bond-paths (BP) with bond critical points (BCP in red) between the atom critical points (ACP, numbering shown in black). The colour of the bond-paths changes according to electron density, from blue (high density) to green to red (low density). Ring critical points (RCP) are shown in green. QTAIM determined electron density ρ at the BCP of the intra-molecular BP between the triazole N and a nearby pyridine H (CP67 and CP82) is 0.0049 e a_0_^–3^ (see Tables 6 and 7 for more values). Colour code of atoms (online version): Fe (red), Cl (green), N (blue), C (black), H (white).

Data of a quantum theory of atoms in molecules (QTAIM) study of dichloro{bis[2-(1-phenyl-1H-1,2,3-triazol-4-yl-κN^3^]pyridine-κN]}iron(II), [Fe^II^(L^8^)_2_Cl_2_], are given in Tables 6 and 7. QTAIM uses the electron density of molecules as a tool to characterize the strength of bonds in various molecular systems [4]. Bonds between atoms are identified by bond paths (BP) between atoms (atom critical points ACP), with bond critical points (BCP) [5]. The total number of critical points is 133 that includes 66 bond critical points (3,−1), 10 ring critical points (3,+1) and 57 atom critical points (3,-3). QTAIM identified a bond path (BP) between a triazole N and a nearby pyridine H, stabilizing the *trans-trans-trans* isomer, see Fig. 6. This bond path stabilizes the *trans* orientation of the triazol-pyridine ligand in *trans-trans-trans* dichloro{bis[2-(1-phenyl-1H-1,2,3-triazol-4-yl-κN^3^]pyridine-κN]}iron(II), [Fe^II^(L^8^)_2_Cl_2_].

**Table 6:** QTAIM results for the 57 ACP (3,-3) of Dichloro{bis[2-(1-phenyl-1H-1,2,3-triazol-4-yl-κN^3^]pyridine-κN]}iron(II), [Fe^II^(L^8^)_2_Cl_2_] (containing L^8^ with R = H). Atom numbering is indicated in Fig. 6.

CP number	*ρ*/*e a*_0_^–3^	CP number	*ρ*/*e a*_0_^–3^	CP number	*ρ*/*e a*_0_^–3^
1	1.19E+04	21	1.26E+02	41	5.08E-01
2	3.21E+03	22	1.26E+02	42	5.02E-01
3	3.21E+03	23	1.26E+02	43	5.08E-01
4	2.05E+02	24	1.26E+02	44	5.08E-01
5	2.05E+02	25	1.26E+02	45	5.12E-01
6	2.05E+02	26	1.26E+02	46	5.08E-01
7	2.04E+02	27	1.26E+02	47	5.02E-01
8	2.05E+02	28	1.26E+02	48	5.08E-01
9	2.05E+02	29	1.26E+02	49	5.06E-01
10	2.05E+02	30	1.26E+02	50	5.10E-01
11	2.04E+02	31	1.26E+02	51	5.10E-01
12	1.26E+02	32	1.26E+02	52	5.10E-01
13	1.26E+02	33	1.26E+02	53	5.08E-01
14	1.26E+02	34	1.26E+02	54	5.06E-01
15	1.26E+02	35	1.26E+02	55	5.10E-01
16	1.26E+02	36	1.26E+02	56	5.10E-01
17	1.26E+02	37	1.26E+02	57	5.10E-01
18	1.26E+02	38	5.08E-01		
19	1.26E+02	39	5.08E-01		
20	1.26E+02	40	5.12E-01

**Table 7:** QTAIM results for the BCP (3,-1) and RCP (3,+1) of dichloro{bis[2-(1-phenyl-1H-1,2,3-triazol-4-yl-κN^3^]pyridine-κN]}iron(II), [Fe^II^(L^8^)_2_Cl_2_] (containing L^8^ with R = H). Atom numbering is indicated in Fig. 6.

CP No.	RANK, SIGNATURE	type	ρ/e a_0_^–3^	∇^2^ρ/e a_0_^–5^	BP No.	Atom	Atom	Distance	BP length	BP Steps
58	(3,−1)	BCP	3.08E-01	−0.8188	62	50	52	1.393	1.393	21
59	(3,−1)	BCP	3.07E-01	−0.8166	64	51	52	1.394	1.394	21
60	(3,−1)	BCP	3.08E-01	−0.8221	60	49	51	1.391	1.391	22
61	(3,+1)	RCP	2.37E-02	0.1507						
62	(3,−1)	BCP	2.81E-01	−0.9760	65	51	56	1.084	1.084	17
63	(3,−1)	BCP	2.73E-01	−0.7033	33	23	47	1.426	1.426	21
64	(3,−1)	BCP	2.85E-01	−1.0172	44	34	35	1.075	1.075	22
65	(3,−1)	BCP	3.18E-01	−0.8463	43	33	34	1.376	1.377	24
66	(3,+1)	RCP	3.43E-03	0.0152						
67	(3,−1)	BCP	4.85E-03	0.0185	17	8	22	2.743	2.767	33
68	(3,+1)	RCP	2.54E-02	0.1665						
69	(3,−1)	BCP	3.37E-01	−0.9744	28	20	32	1.345	1.345	20
70	(3,−1)	BCP	4.93E-02	0.2223	6	1	21	2.243	2.246	29
71	(3,−1)	BCP	5.02E-02	0.2192	5	1	20	2.253	2.254	28
72	(3,−1)	BCP	5.59E-02	0.1679	1	1	2	2.399	2.399	27
73	(3,−1)	BCP	5.59E-02	0.1679	4	1	19	2.399	2.399	28
74	(3,+1)	RCP	1.87E-02	0.0943						
75	(3,−1)	BCP	5.02E-02	0.2192	2	1	3	2.253	2.254	26
76	(3,−1)	BCP	3.40E-01	−0.9938	27	20	24	1.334	1.335	21
77	(3,+1)	RCP	3.43E-03	0.0152						
78	(3,+1)	RCP	1.87E-02	0.0943						
79	(3,−1)	BCP	2.80E-01	−0.9755	36	26	27	1.083	1.083	17
80	(3,−1)	BCP	2.73E-01	−0.6858	24	15	16	1.465	1.465	24
81	(3,−1)	BCP	3.24E-01	−0.8846	10	4	16	1.366	1.367	25
82	(3,−1)	BCP	4.85E-03	0.0185	12	5	25	2.743	2.767	35
83	(3,+1)	RCP	5.88E-02	0.4267						
84	(3,−1)	BCP	3.18E-01	−0.8463	25	16	17	1.376	1.377	24
85	(3,−1)	BCP	3.62E-01	−0.6030	11	5	6	1.351	1.351	23
86	(3,−1)	BCP	2.73E-01	−0.7033	14	6	36	1.426	1.426	20
87	(3,−1)	BCP	3.08E-01	−0.8221	49	38	40	1.391	1.391	22
88	(3,+1)	RCP	2.37E-02	0.1507						
89	(3,−1)	BCP	3.08E-01	−0.8165	45	36	37	1.394	1.394	21
90	(3,−1)	BCP	2.80E-01	−0.9675	48	37	42	1.083	1.083	17
91	(3,−1)	BCP	3.07E-01	−0.8171	47	37	39	1.392	1.392	17
92	(3,−1)	BCP	3.08E-01	−0.8188	51	39	41	1.393	1.393	17
93	(3,−1)	BCP	2.80E-01	−0.9736	52	39	44	1.084	1.084	15
94	(3,−1)	BCP	2.80E-01	−0.9704	66	52	57	1.084	1.084	17
95	(3,−1)	BCP	2.80E-01	−0.9736	63	50	55	1.084	1.084	15
96	(3,−1)	BCP	3.07E-01	−0.8171	58	48	50	1.392	1.392	17
97	(3,−1)	BCP	3.08E-01	−0.8165	56	47	48	1.394	1.394	17
98	(3,−1)	BCP	3.08E-01	−0.8182	57	47	49	1.394	1.395	17
99	(3,−1)	BCP	3.12E-01	−0.7856	32	23	34	1.360	1.360	21
100	(3,+1)	RCP	5.88E-02	0.4267						
101	(3,−1)	BCP	3.10E-01	−0.8304	39	28	30	1.389	1.389	20
102	(3,+1)	RCP	2.54E-02	0.1665						
103	(3,−1)	BCP	3.09E-01	−0.8281	19	9	11	1.391	1.391	23
104	(3,−1)	BCP	3.09E-01	−0.8281	37	26	28	1.391	1.391	21
105	(3,−1)	BCP	4.93E-02	0.2223	3	1	4	2.243	2.246	25
106	(3,−1)	BCP	2.79E-01	−0.9647	22	13	14	1.083	1.083	17
107	(3,−1)	BCP	3.12E-01	−0.7856	13	6	17	1.360	1.360	20
108	(3,−1)	BCP	2.85E-01	−1.0172	26	17	18	1.075	1.075	18
109	(3,−1)	BCP	2.83E-01	−0.9950	50	38	43	1.082	1.082	17
110	(3,−1)	BCP	3.62E-01	−0.6030	31	22	23	1.351	1.351	19
111	(3,−1)	BCP	4.17E-01	−0.8808	29	21	22	1.292	1.295	19
112	(3,−1)	BCP	2.79E-01	−0.9647	40	30	31	1.083	1.083	16
113	(3,−1)	BCP	2.73E-01	−0.6858	42	32	33	1.465	1.465	19
114	(3,-1)	BCP	3.08E-01	−0.8201	41	30	32	1.396	1.396	18
115	(3,−1)	BCP	2.87E-01	−1.0384	15	7	8	1.084	1.084	17
116	(3,−1)	BCP	3.12E-01	−0.8441	35	24	26	1.391	1.391	18
117	(3,−1)	BCP	3.10E-01	−0.8304	21	11	13	1.389	1.389	18
118	(3,−1)	BCP	3.08E-01	−0.8201	23	13	15	1.396	1.396	18
119	(3,−1)	BCP	2.80E-01	−0.9704	55	41	46	1.084	1.084	16
120	(3,−1)	BCP	2.83E-01	−0.9950	61	49	54	1.082	1.082	17
121	(3,−1)	BCP	3.24E-01	−0.8846	30	21	33	1.366	1.367	17
122	(3,−1)	BCP	3.12E-01	−0.8441	16	7	9	1.391	1.391	18
123	(3,-1)	BCP	3.40E-01	−0.9938	7	3	7	1.334	1.335	18
124	(3,−1)	BCP	3.37E-01	−0.9744	8	3	15	1.345	1.345	19
125	(3,−1)	BCP	2.87E-01	−1.0384	34	24	25	1.084	1.084	16
126	(3,−1)	BCP	4.17E-01	−0.8808	9	4	5	1.292	1.295	17
127	(3,−1)	BCP	3.08E-01	−0.8182	46	36	38	1.394	1.395	17
128	(3,−1)	BCP	2.80E-01	−0.9675	59	48	53	1.083	1.083	16
129	(3,−1)	BCP	3.07E-01	−0.8166	53	40	41	1.394	1.394	17
130	(3,−1)	BCP	2.81E-01	−0.9760	54	40	45	1.084	1.084	17
131	(3,−1)	BCP	2.80E-01	−0.9755	18	9	10	1.083	1.083	15
132	(3,−1)	BCP	2.80E-01	−0.9707	38	28	29	1.084	1.084	17
133	(3,−1)	BCP	2.80E-01	−0.9707	20	11	12	1.084	1.084	15

**Fig. 7:**
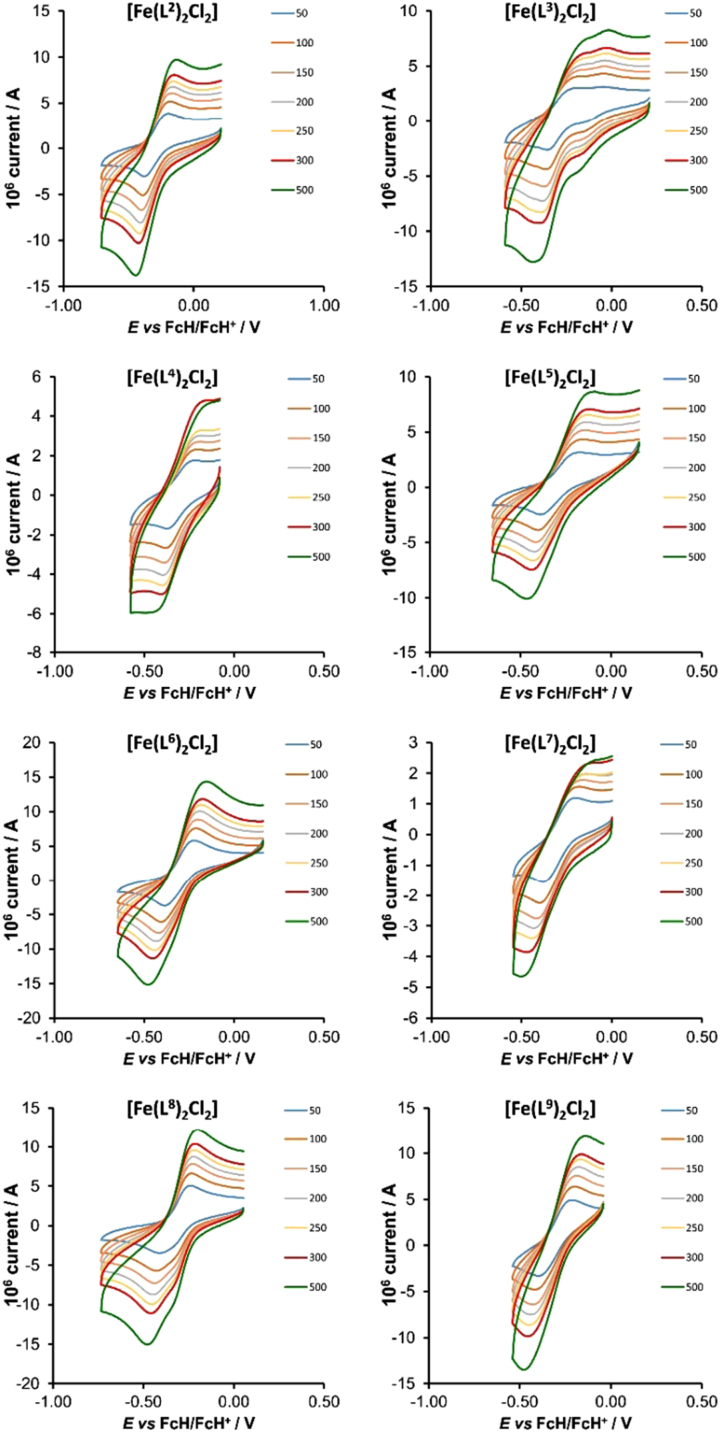
Cyclic voltammograms of the Fe^II/III^ redox couple of dichloro(bis{2-[1-(4-R-phenyl)-1H-1,2,3-triazol-4-yl-κN^3^]pyridine-κN})iron(II) coordination compounds [Fe(L^n^)_2_Cl_2_ at scan rates of 0.05 (lowest peak current) – 0.50 (highest peak current) Vs^-1^ (0.10 Vs^-1^ scans from [1]). All scans initiated in the positive direction. R=CH_3_ (L^2^), OCH_3_ (L^3^), COOH (L^4^), F (L^5^), Cl (L^6^), CN (L^7^), H (L^8^) and CF_3_ (L^9^). Values *E*°׳ (V *vs.* Fc/Fc^+^) at 0.100 V s^-1^=−0.287, −0.282, −0.296, −0.281, −0.311, −0.295, −0.333 and −0.310 V [1], for complexes 2 to 9 respectively. CV׳s obtained in 1:3 acetonitrile/DMSO solution with 0.1 mol dm^−3^ (^n^Bu_4_N)(PF_6_) as supporting electrolyte.

Cyclic voltammograms of the Fe^II/III^ redox couple of dichloro(bis{2-[1-(4-R-phenyl)-1H-1,2,3-triazol-4-yl-κN^3^]pyridine-κN})iron(II) coordination compounds [Fe(L^n^)_2_Cl_2_ (n = 2-9) at scan rates of 0.05–0.50 Vs^−1^ are shown in Fig. 7 (0.10 Vs^-1^ scans from reference [1]). The formal reduction potentials, calculated as *E*°′ = (*E*_pa_ + *E*_pc_)/2 with *E*_pa_ = peak anodic potential and *E*_pc_ = peak cathodic potential, varies from −0.287 to −0.310 V *vs.* Fc/Fc^+^, for the different [Fe(L^n^)_2_Cl_2_ (n = 2-9) compounds. The near similar values of the formal reduction potentials of *ca* −0.3 V obtained for the different complexes, suggest that the electronic properties of the different R substituents on the phenyl groups does not have an observable influence on the charge of Fe(II) in the different Fe(L^n^)_2_Cl_2_ complexes.

## 2 Experimental design, materials and methods

Density functional theory (DFT) calculations were performed in the gas phase on the neutral compounds, using the B3LYP functional and the triple-ζ basis set 6–311 G(d,p) on all atoms. The Gaussian 09 package [6] were used to optimize the compounds. The multiplicity used for the Fe compounds is quintet (high spin with S = 2, i.e. 4 unpaired electrons). Natural bonding orbital (NBO) calculations [7], [8], [9], [10] were performed on the optimised structure of [Fe^II^(L^8^)_2_Cl_2_] using the NBO 3.1 module [11] in Gaussian 09, at the same level of theory. Quantum theory of atoms in molecules (QTAIM) calculations have been performed on the optimised structure of [Fe^II^(L^8^)_2_Cl_2_] using Bader׳s QTAIM [12], [13], [14]. Cyclic voltammetry measurements [15] were obtained on a BAS 100B/W electrochemical analyzer, on 0.0005 mmol dm^-3^ or saturated compound solutions, in a dry 3:1 acetonitrile/DMSO solution (Aldrich, Biotech grade 99.93+% purity, anhydrous, kept under purified argon), using 0.1 mol dm^-3^ tetra-*n*-butylammonium hexafluorophosphate, (^n^Bu_4_N)(PF_6_) (Fluka electrochemical grade) as supporting electrolyte. A three-electrode cell, with a glassy carbon (surface area 7.07 × 10^-6^ m^2^) working electrode, Pt auxiliary electrode and a Ag/Ag^+^ (0.010 mol dm^-3^ AgNO_3_ in CH_3_CN) reference electrode [16], mounted on a Luggin capillary, was used [17], [18]. The analyte and electrochemical cell were kept at 25 °C under a blanket of purified argon during the cyclic voltammetry experiment. All cited potentials were referenced against the Fc/Fc^+^ couple, as suggested by IUPAC [19].
