# Supplementary material for: DFT and CV data of 4-phenyl-substituted dichloro(bis{2-[1-(phenyl)-1H-1,2,3-triazol-4-yl-κN3]pyridine-κN})iron(II) coordination compounds
Source: Data Brief. 2018 Oct 27;21:1458–71. doi: 10.1016/j.dib.2018.10.085 (PMC6234228; doi:10.1016/j.dib.2018.10.085)
Supplement: Supplementary file 3 — Supplementary material. [file mmc3.docx]

**Data Article**

**Title**: DFT and CV data of 4-phenyl-substituted Dichloro(bis{2-[1-(phenyl)-1H-1,2,3-triazol-4-yl-κN^3^]pyridine-κN})iron(II) coordination compounds

**Authors**: J. Conradie^1*^, M.M. Conradie^1^, Z. Mtshali^1^ and J.H. Potgieter^2,3*^

**Affiliations**:

1. Department of Chemistry, University of the Free State, P.O. Box 339, Bloemfontein, 9300, South Africa

2. Division of Chemistry and Environmental Science, Manchester Metropolitan University, Manchester, M1 5GD, UK

3. School of Chemical and Metallurgical Engineering, University of the Witwatersrand, Private Bag X3, Wits, 2050, South Africa

**Contact email**: conradj@ufs.ac.za

Supporting information

Table of Contents

[Optimized Cartesian coordinates (Å) 2](#_Toc527764702)

[1. [Fe(L^2^)_2_Cl_2_] with R = CH_3_ 2](#_Toc527764703)

[2. [Fe(L^3^)_2_Cl_2_] with R = OCH_3_ 3](#_Toc527764704)

[3. [Fe(L^4^)_2_Cl_2_] with R = COOH 5](#_Toc527764705)

[4. [Fe(L^5^)_2_Cl_2_] with R = F 6](#_Toc527764706)

[5. [Fe(L^6^)_2_Cl_2_] with R = Cl 7](#_Toc527764707)

[6. [Fe(L^7^)_2_Cl_2_] with R = CN 8](#_Toc527764708)

[7. [Fe(L^8^)_2_Cl_2_] with R = H 10](#_Toc527764709)

[8. [Fe(L^9^)_2_Cl_2_] with R = CF_3_ 11](#_Toc527764710)

# Optimized Cartesian coordinates (Å)

All compounds were optimized as a quintet using the B3LYP functional as implemented in the Gaussian 09 package [^^[[1]](#endnote-1)^^] using the triple-ζ basis set 6-311G(d,p).

Figure 1 Structure of the dichloro(bis{2-[1-(4-R-phenyl)-1H-1,2,3-triazol-4-yl-κN^3^]pyridine-κN})iron(II), [Fe(L^n^)_2_Cl_2_], compounds. R = CH_3_ (L^2^), OCH_3_ (L^3^), COOH (L^4^), F (L^5^), Cl (L^6^), CN (L^7^), H (L^8^) and CF_3_ (L^9^).

# [Fe(L^2^)_2_Cl_2_] with R = CH_3_

Fe -0.000005000 -0.000036000 -0.000949000

Cl 0.033220000 0.147533000 2.394981000

N 0.115928000 -2.247220000 0.121493000

N 2.186235000 -0.490458000 0.008250000

N 3.285851000 0.188254000 -0.020221000

N 4.299401000 -0.703424000 0.006425000

C -0.948154000 -3.048707000 0.185594000

H -1.912835000 -2.554028000 0.191674000

C -0.840345000 -4.434233000 0.242612000

H -1.731183000 -5.047173000 0.295473000

C 0.429734000 -5.002469000 0.229978000

H 0.553940000 -6.078440000 0.271650000

C 1.541414000 -4.172337000 0.163912000

H 2.541798000 -4.587326000 0.152784000

C 1.345777000 -2.791062000 0.112451000

C 2.448923000 -1.829791000 0.052603000

C 3.818078000 -1.974265000 0.052394000

H 4.464737000 -2.831205000 0.111892000

Cl -0.033236000 -0.147585000 -2.396659000

N -0.115939000 2.247187000 -0.122801000

N -2.186235000 0.490421000 -0.009488000

N -3.285853000 -0.188276000 0.019250000

N -4.299390000 0.703452000 -0.006210000

C 0.948129000 3.048675000 -0.187111000

H 1.912796000 2.553974000 -0.193914000

C 0.840328000 4.434230000 -0.243428000

H 1.731154000 5.047171000 -0.296482000

C -0.429728000 5.002495000 -0.229842000

H -0.553927000 6.078489000 -0.270944000

C -1.541396000 4.172362000 -0.163572000

H -2.541763000 4.587370000 -0.151726000

C -1.345768000 2.791058000 -0.112854000

C -2.448909000 1.829789000 -0.052884000

C -3.818059000 1.974305000 -0.051733000

H -4.464735000 2.831297000 -0.110285000

C 5.656288000 -0.264035000 0.001336000

C 6.614870000 -0.969562000 -0.720628000

C 6.009536000 0.876306000 0.721353000

C 7.937227000 -0.536097000 -0.705489000

C 7.331402000 1.301763000 0.712044000

C 8.319892000 0.602880000 0.008006000

H 6.328998000 -1.830683000 -1.312599000

H 5.251008000 1.412443000 1.276099000

H 8.679071000 -1.086360000 -1.273859000

H 7.601551000 2.191078000 1.271481000

C -5.656288000 0.264100000 -0.000450000

C -6.614310000 0.969105000 0.722708000

C -6.010098000 -0.875690000 -0.721117000

C -7.936722000 0.535686000 0.708178000

C -7.331946000 -1.301092000 -0.711204000

C -8.319924000 -0.602723000 -0.005871000

H -6.328004000 1.829802000 1.315084000

H -5.251988000 -1.411393000 -1.276854000

H -8.678145000 1.085581000 1.277445000

H -7.602534000 -2.189937000 -1.271189000

C 9.757280000 1.060608000 0.036256000

H 10.334607000 0.612074000 -0.774377000

H 9.830469000 2.147044000 -0.056072000

H 10.238309000 0.780865000 0.979528000

C -9.757216000 -1.060814000 -0.033161000

H -10.335896000 -0.606577000 0.773298000

H -10.236748000 -0.788111000 -0.979230000

H -9.830361000 -2.146597000 0.066960000

# [Fe(L^3^)_2_Cl_2_] with R = OCH_3_

Fe 0.000000000 0.000000000 0.000000000

Cl -1.910747000 -0.374248000 1.407710000

O 4.937946000 2.121550000 8.032062000

N -0.627891000 2.143055000 -0.302747000

N 0.967437000 1.148941000 1.659924000

N 1.706142000 0.854395000 2.679773000

N 1.940493000 2.007150000 3.341834000

C -1.421263000 2.559922000 -1.290668000

H -1.788213000 1.789911000 -1.959962000

C -1.765469000 3.896607000 -1.462241000

H -2.414707000 4.190441000 -2.277274000

C -1.257326000 4.833346000 -0.567548000

H -1.501314000 5.884456000 -0.670285000

C -0.431447000 4.404771000 0.463555000

H -0.021540000 5.111946000 1.174226000

C -0.138277000 3.043765000 0.567787000

C 0.707058000 2.488333000 1.626421000

C 1.333814000 3.050456000 2.716487000

H 1.370413000 4.052021000 3.106058000

C 2.704342000 2.013704000 4.545955000

C 2.531594000 0.991981000 5.472649000

H 1.818234000 0.204225000 5.269377000

C 3.270642000 0.989357000 6.652435000

H 3.120997000 0.186608000 7.360944000

C 4.176988000 2.022912000 6.911999000

C 4.342765000 3.049048000 5.971526000

H 5.059789000 3.832229000 6.182630000

C 3.618671000 3.040828000 4.791675000

H 3.779398000 3.816666000 4.052747000

C 4.828939000 1.106149000 9.023595000

H 5.110925000 0.126119000 8.624385000

H 3.815360000 1.056690000 9.435478000

H 5.524468000 1.387922000 9.811728000

Cl 1.910747000 0.374248000 -1.407710000

O -4.937946000 -2.121550000 -8.032062000

N 0.627891000 -2.143055000 0.302747000

N -0.967437000 -1.148941000 -1.659924000

N -1.706142000 -0.854395000 -2.679773000

N -1.940493000 -2.007150000 -3.341834000

C 1.421263000 -2.559922000 1.290668000

H 1.788213000 -1.789911000 1.959962000

C 1.765469000 -3.896607000 1.462241000

H 2.414707000 -4.190441000 2.277274000

C 1.257326000 -4.833346000 0.567548000

H 1.501314000 -5.884456000 0.670285000

C 0.431447000 -4.404771000 -0.463555000

H 0.021540000 -5.111946000 -1.174226000

C 0.138277000 -3.043765000 -0.567787000

C -0.707058000 -2.488333000 -1.626421000

C -1.333814000 -3.050456000 -2.716487000

H -1.370413000 -4.052021000 -3.106058000

C -2.704342000 -2.013704000 -4.545955000

C -2.531594000 -0.991981000 -5.472649000

H -1.818234000 -0.204225000 -5.269377000

C -3.270642000 -0.989357000 -6.652435000

H -3.120997000 -0.186608000 -7.360944000

C -4.176988000 -2.022912000 -6.911999000

C -4.342765000 -3.049048000 -5.971526000

H -5.059789000 -3.832229000 -6.182630000

C -3.618671000 -3.040828000 -4.791675000

H -3.779398000 -3.816666000 -4.052747000

C -4.828939000 -1.106149000 -9.023595000

H -5.110925000 -0.126119000 -8.624385000

H -3.815360000 -1.056690000 -9.435478000

H -5.524468000 -1.387922000 -9.811728000

# [Fe(L^4^)_2_Cl_2_] with R = COOH

Fe 0.000001000 0.000003000 0.000056000

Cl 0.009360000 0.163643000 2.387532000

N 0.026130000 -2.251681000 0.142398000

N 2.168551000 -0.581074000 0.036133000

N 3.293073000 0.050831000 0.008710000

N 4.270703000 -0.883135000 0.039141000

C -1.069791000 -3.009155000 0.206102000

H -2.014233000 -2.477112000 0.199247000

C -1.019055000 -4.397316000 0.278497000

H -1.934608000 -4.972686000 0.330255000

C 0.226498000 -5.017025000 0.282686000

H 0.306481000 -6.096510000 0.336972000

C 1.371457000 -4.233164000 0.217322000

H 2.353923000 -4.689068000 0.219879000

C 1.232220000 -2.846161000 0.149266000

C 2.374814000 -1.931819000 0.087804000

C 3.735302000 -2.133986000 0.088832000

H 4.342642000 -3.018769000 0.152101000

Cl -0.009358000 -0.163637000 -2.387421000

N -0.026131000 2.251689000 -0.142276000

N -2.168549000 0.581078000 -0.036033000

N -3.293070000 -0.050832000 -0.008657000

N -4.270703000 0.883130000 -0.039134000

C 1.069789000 3.009167000 -0.205948000

H 2.014232000 2.477125000 -0.199096000

C 1.019051000 4.397329000 -0.278311000

H 1.934604000 4.972702000 -0.330044000

C -0.226503000 5.017036000 -0.282497000

H -0.306486000 6.096523000 -0.336756000

C -1.371461000 4.233172000 -0.217165000

H -2.353927000 4.689074000 -0.219717000

C -1.232222000 2.846168000 -0.149144000

C -2.374815000 1.931822000 -0.087718000

C -3.735303000 2.133984000 -0.088807000

H -4.342644000 3.018764000 -0.152109000

C 5.640008000 -0.497375000 0.027616000

C 6.582451000 -1.305902000 -0.608202000

C 6.018572000 0.690216000 0.656535000

C 7.919457000 -0.929600000 -0.602610000

C 7.353743000 1.063501000 0.645297000

C 8.312314000 0.257114000 0.022927000

H 6.272029000 -2.204418000 -1.126576000

H 5.268792000 1.296610000 1.145672000

H 8.658575000 -1.546306000 -1.095569000

H 7.676576000 1.977750000 1.126786000

C -5.640006000 0.497366000 -0.027669000

C -6.582484000 1.305900000 0.608087000

C -6.018534000 -0.690239000 -0.656585000

C -7.919489000 0.929593000 0.602434000

C -7.353705000 -1.063529000 -0.645408000

C -8.312310000 -0.257134000 -0.023099000

H -6.272092000 2.204427000 1.126460000

H -5.268728000 -1.296639000 -1.145675000

H -8.658633000 1.546305000 1.095346000

H -7.676510000 -1.977787000 -1.126896000

C 9.730487000 0.708291000 0.049629000

O 10.118352000 1.726541000 0.568040000

O 10.566292000 -0.152955000 -0.578418000

H 11.453739000 0.228172000 -0.509124000

C -9.730479000 -0.708319000 -0.049862000

O -10.118324000 -1.726546000 -0.568332000

O -10.566329000 0.152977000 0.578058000

H -11.453778000 -0.228128000 0.508681000

# [Fe(L^5^)_2_Cl_2_] with R = F

Fe 0.000000000 0.000000000 0.000000000

Cl 0.034143000 0.150752000 2.392417000

N 0.125315000 -2.248281000 0.124372000

N 2.189017000 -0.484097000 0.009326000

N 3.285233000 0.199133000 -0.018772000

N 4.302439000 -0.688950000 0.008558000

C -0.935437000 -3.053991000 0.189938000

H -1.902074000 -2.563250000 0.197296000

C -0.822419000 -4.439146000 0.246897000

H -1.710928000 -5.055303000 0.301100000

C 0.449533000 -5.002852000 0.232506000

H 0.577755000 -6.078290000 0.273980000

C 1.558002000 -4.168381000 0.165017000

H 2.559838000 -4.579806000 0.152597000

C 1.356867000 -2.788101000 0.113950000

C 2.456689000 -1.822673000 0.053630000

C 3.826258000 -1.962176000 0.054272000

H 4.475875000 -2.816905000 0.115071000

Cl -0.034143000 -0.150752000 -2.392417000

N -0.125315000 2.248281000 -0.124372000

N -2.189017000 0.484097000 -0.009326000

N -3.285233000 -0.199133000 0.018772000

N -4.302439000 0.688950000 -0.008558000

C 0.935437000 3.053991000 -0.189939000

H 1.902074000 2.563250000 -0.197296000

C 0.822419000 4.439146000 -0.246897000

H 1.710928000 5.055303000 -0.301100000

C -0.449533000 5.002852000 -0.232506000

H -0.577755000 6.078290000 -0.273980000

C -1.558002000 4.168381000 -0.165017000

H -2.559838000 4.579806000 -0.152597000

C -1.356867000 2.788100000 -0.113950000

C -2.456689000 1.822673000 -0.053630000

C -3.826258000 1.962176000 -0.054272000

H -4.475875000 2.816905000 -0.115071000

C 5.655624000 -0.241282000 0.005107000

C 6.613158000 -0.927414000 -0.740383000

C 6.001444000 0.885987000 0.750470000

C 7.934704000 -0.494013000 -0.729551000

C 7.317853000 1.330569000 0.748960000

C 8.263757000 0.629723000 0.013778000

H 6.327518000 -1.778640000 -1.345732000

H 5.241449000 1.400542000 1.322795000

H 8.700434000 -1.002781000 -1.300664000

H 7.618964000 2.201068000 1.317442000

C -5.655624000 0.241282000 -0.005107000

C -6.613158000 0.927414000 0.740384000

C -6.001444000 -0.885987000 -0.750470000

C -7.934704000 0.494013000 0.729552000

C -7.317853000 -1.330569000 -0.748960000

C -8.263757000 -0.629723000 -0.013778000

H -6.327518000 1.778640000 1.345732000

H -5.241449000 -1.400542000 -1.322794000

H -8.700434000 1.002781000 1.300664000

H -7.618964000 -2.201068000 -1.317442000

F 9.541862000 1.055494000 0.019990000

F -9.541862000 -1.055494000 -0.019989000

# [Fe(L^6^)_2_Cl_2_] with R = Cl

Fe 0.000000000 0.000000000 0.000000000

Cl 0.037464000 -0.146700000 -2.390539000

N 0.053732000 2.252629000 -0.122233000

N 2.173192000 0.554848000 -0.010090000

N 3.290228000 -0.092617000 0.017120000

N 4.278877000 0.828135000 -0.006644000

C -1.031993000 3.024494000 -0.187094000

H -1.982874000 2.503958000 -0.194258000

C -0.963142000 4.412584000 -0.243616000

H -1.870837000 5.000125000 -0.297382000

C 0.290154000 5.016479000 -0.229537000

H 0.384185000 6.095427000 -0.270840000

C 1.424536000 4.217536000 -0.162648000

H 2.412715000 4.660761000 -0.150620000

C 1.267342000 2.831650000 -0.111847000

C 2.397566000 1.901945000 -0.051916000

C 3.761524000 2.085791000 -0.050008000

H 4.382416000 2.961709000 -0.108230000

Cl -0.037464000 0.146699000 2.390540000

N -0.053732000 -2.252629000 0.122234000

N -2.173192000 -0.554848000 0.010091000

N -3.290227000 0.092617000 -0.017120000

N -4.278877000 -0.828135000 0.006644000

C 1.031993000 -3.024494000 0.187094000

H 1.982874000 -2.503958000 0.194259000

C 0.963141000 -4.412584000 0.243617000

H 1.870837000 -5.000125000 0.297382000

C -0.290154000 -5.016479000 0.229537000

H -0.384186000 -6.095427000 0.270840000

C -1.424536000 -4.217536000 0.162649000

H -2.412715000 -4.660761000 0.150620000

C -1.267342000 -2.831650000 0.111848000

C -2.397566000 -1.901945000 0.051916000

C -3.761524000 -2.085791000 0.050008000

H -4.382416000 -2.961709000 0.108229000

C 5.644383000 0.423981000 -0.001418000

C 6.586906000 1.163268000 0.710619000

C 6.021346000 -0.714558000 -0.712443000

C 7.921314000 0.771773000 0.700319000

C 7.351851000 -1.114734000 -0.710546000

C 8.292695000 -0.365430000 -0.009444000

H 6.282531000 2.024482000 1.292372000

H 5.275340000 -1.273444000 -1.261127000

H 8.662153000 1.335203000 1.251738000

H 7.658814000 -1.995295000 -1.259137000

C -5.644383000 -0.423981000 0.001418000

C -6.586906000 -1.163268000 -0.710619000

C -6.021346000 0.714558000 0.712443000

C -7.921314000 -0.771773000 -0.700319000

C -7.351851000 1.114734000 0.710545000

C -8.292695000 0.365430000 0.009444000

H -6.282531000 -2.024482000 -1.292372000

H -5.275341000 1.273444000 1.261127000

H -8.662153000 -1.335203000 -1.251739000

H -7.658814000 1.995295000 1.259137000

Cl 9.974182000 -0.865541000 -0.017449000

Cl -9.974182000 0.865541000 0.017449000

# [Fe(L^7^)_2_Cl_2_] with R = CN

Fe 0.000000000 0.000000000 0.000000000

Cl 0.027186000 0.140690000 2.385026000

N 0.070800000 -2.254166000 0.117317000

N 2.181208000 -0.543010000 0.014502000

N 3.292904000 0.110129000 -0.007675000

N 4.288429000 -0.806187000 0.009007000

C -1.010092000 -3.032871000 0.180088000

H -1.964056000 -2.518245000 0.189397000

C -0.933189000 -4.420716000 0.232288000

H -1.837369000 -5.013743000 0.284452000

C 0.323342000 -5.017534000 0.216121000

H 0.423556000 -6.095961000 0.254018000

C 1.453011000 -4.211601000 0.151807000

H 2.443608000 -4.649299000 0.138654000

C 1.287313000 -2.826775000 0.105343000

C 2.412658000 -1.890658000 0.049029000

C 3.776384000 -2.067869000 0.044224000

H 4.399289000 -2.942577000 0.096446000

Cl -0.027186000 -0.140691000 -2.385026000

N -0.070800000 2.254166000 -0.117317000

N -2.181208000 0.543010000 -0.014502000

N -3.292904000 -0.110129000 0.007675000

N -4.288429000 0.806187000 -0.009007000

C 1.010092000 3.032871000 -0.180088000

H 1.964056000 2.518245000 -0.189397000

C 0.933189000 4.420716000 -0.232289000

H 1.837369000 5.013743000 -0.284453000

C -0.323342000 5.017533000 -0.216121000

H -0.423556000 6.095961000 -0.254019000

C -1.453011000 4.211601000 -0.151808000

H -2.443608000 4.649299000 -0.138655000

C -1.287313000 2.826775000 -0.105343000

C -2.412658000 1.890658000 -0.049029000

C -3.776384000 2.067869000 -0.044224000

H -4.399289000 2.942577000 -0.096446000

C 5.647756000 -0.391917000 0.002795000

C 6.610399000 -1.171961000 -0.638525000

C 5.999190000 0.797833000 0.642537000

C 7.937639000 -0.767575000 -0.628021000

C 7.323877000 1.206597000 0.639789000

C 8.303819000 0.424986000 0.010344000

H 6.323908000 -2.073279000 -1.165307000

H 5.235556000 1.383142000 1.135727000

H 8.691351000 -1.364166000 -1.125537000

H 7.608334000 2.126273000 1.134726000

C -5.647756000 0.391918000 -0.002794000

C -6.610398000 1.171961000 0.638525000

C -5.999190000 -0.797833000 -0.642537000

C -7.937639000 0.767576000 0.628021000

C -7.323877000 -1.206597000 -0.639788000

C -8.303819000 -0.424986000 -0.010344000

H -6.323908000 2.073279000 1.165308000

H -5.235557000 -1.383142000 -1.135727000

H -8.691351000 1.364166000 1.125537000

H -7.608334000 -2.126273000 -1.134725000

C -9.671383000 -0.843934000 -0.016636000

N -10.775954000 -1.181118000 -0.022034000

C 9.671383000 0.843934000 0.016637000

N 10.775954000 1.181118000 0.022035000

# [Fe(L^8^)_2_Cl_2_] with R = H

Fe 0.000000000 0.000000000 -0.000004000

Cl 0.014072000 -0.150572000 -2.394294000

N 0.229714000 2.238087000 -0.124274000

N 2.210380000 0.380686000 -0.015808000

N 3.274498000 -0.351633000 0.009920000

N 4.331370000 0.488973000 -0.012148000

C -0.793248000 3.091461000 -0.188646000

H -1.781200000 2.645155000 -0.198370000

C -0.616916000 4.470142000 -0.241853000

H -1.476256000 5.126504000 -0.294761000

C 0.679558000 4.974981000 -0.225305000

H 0.856788000 6.043590000 -0.263717000

C 1.748702000 4.090640000 -0.159925000

H 2.768316000 4.455810000 -0.146321000

C 1.484783000 2.720716000 -0.112490000

C 2.539175000 1.705921000 -0.054814000

C 3.913483000 1.782578000 -0.053058000

H 4.601313000 2.607115000 -0.108349000

Cl -0.014072000 0.150573000 2.394285000

N -0.229714000 -2.238086000 0.124266000

N -2.210380000 -0.380685000 0.015797000

N -3.274498000 0.351633000 -0.009929000

N -4.331370000 -0.488973000 0.012145000

C 0.793248000 -3.091459000 0.188639000

H 1.781201000 -2.645153000 0.198364000

C 0.616917000 -4.470141000 0.241847000

H 1.476257000 -5.126502000 0.294756000

C -0.679557000 -4.974980000 0.225296000

H -0.856787000 -6.043589000 0.263707000

C -1.748701000 -4.090640000 0.159914000

H -2.768315000 -4.455810000 0.146306000

C -1.484783000 -2.720715000 0.112482000

C -2.539175000 -1.705921000 0.054801000

C -3.913482000 -1.782579000 0.053024000

H -4.601313000 -2.607117000 0.108301000

C 5.664448000 -0.018605000 -0.008697000

C 6.658127000 0.650381000 0.704109000

C 5.950849000 -1.181292000 -0.722922000

C 7.958385000 0.153857000 0.687563000

C 7.251257000 -1.674485000 -0.718182000

C 8.257372000 -1.007981000 -0.020388000

H 6.413536000 1.530736000 1.285873000

H 5.160770000 -1.677702000 -1.270206000

H 8.733387000 0.669296000 1.242349000

H 7.479668000 -2.577716000 -1.271493000

H 9.269623000 -1.394579000 -0.025686000

C -5.664448000 0.018604000 0.008710000

C -6.658131000 -0.650373000 -0.704099000

C -5.950846000 1.181283000 0.722950000

C -7.958389000 -0.153851000 -0.687539000

C -7.251255000 1.674474000 0.718224000

C -8.257373000 1.007978000 0.020428000

H -6.413543000 -1.530721000 -1.285875000

H -5.160765000 1.677687000 1.270236000

H -8.733394000 -0.669283000 -1.242327000

H -7.479663000 2.577699000 1.271547000

H -9.269624000 1.394576000 0.025736000

# [Fe(L^9^)_2_Cl_2_] with R = CF_3_

Fe 0.001827000 0.006883000 0.030382000

Cl -0.040538000 0.150345000 -2.357785000

N 0.012259000 -2.246897000 -0.092531000

N -2.157186000 -0.611027000 0.018739000

N -3.292173000 0.002440000 0.042013000

N -4.253702000 -0.947978000 0.014571000

C 1.119842000 -2.987415000 -0.155796000

H 2.055655000 -2.440379000 -0.161487000

C 1.091203000 -4.376863000 -0.213047000

H 2.015517000 -4.937994000 -0.265436000

C -0.144010000 -5.016838000 -0.201622000

H -0.206731000 -6.097987000 -0.243641000

C -1.301014000 -4.250918000 -0.136540000

H -2.275840000 -4.722834000 -0.126672000

C -1.183806000 -2.861221000 -0.084667000

C -2.340999000 -1.965115000 -0.026246000

C -3.698071000 -2.189996000 -0.028210000

H -4.290817000 -3.084893000 -0.089309000

Cl 0.043981000 -0.135719000 2.416908000

N -0.008268000 2.261037000 0.148994000

N 2.160771000 0.625006000 0.034157000

N 3.295660000 0.011706000 0.003174000

N 4.257050000 0.962597000 0.013564000

C -1.115550000 3.001553000 0.217078000

H -2.051190000 2.454341000 0.230293000

C -1.086822000 4.391183000 0.269644000

H -2.010882000 4.952341000 0.326091000

C 0.148179000 5.031324000 0.248282000

H 0.210966000 6.112611000 0.286495000

C 1.304888000 4.265392000 0.178281000

H 2.279553000 4.737425000 0.160936000

C 1.187605000 2.875521000 0.131427000

C 2.344543000 1.979419000 0.067981000

C 3.701486000 2.204710000 0.053735000

H 4.294531000 3.100213000 0.101493000

C -5.629386000 -0.584638000 0.014253000

C -6.560258000 -1.388774000 0.670273000

C -6.025950000 0.578824000 -0.645019000

C -7.903895000 -1.034904000 0.649162000

C -7.368365000 0.932502000 -0.648595000

C -8.309113000 0.124959000 -0.008589000

H -6.238180000 -2.266425000 1.216308000

H -5.285286000 1.187084000 -1.145555000

H -8.632602000 -1.648191000 1.162760000

H -7.686033000 1.836069000 -1.152717000

C 5.632603000 0.599032000 -0.002416000

C 6.554031000 1.397407000 -0.677830000

C 6.039407000 -0.555431000 0.666935000

C 7.898112000 1.043500000 -0.672801000

C 7.381617000 -0.908677000 0.655237000

C 8.313046000 -0.108419000 -0.007859000

H 6.225114000 2.273017000 -1.223077000

H 5.307236000 -1.154339000 1.190730000

H 8.621153000 1.659758000 -1.190690000

H 7.709265000 -1.796843000 1.180110000

C -9.770089000 0.481553000 -0.074040000

F -10.356072000 -0.034089000 -1.178986000

F -10.454316000 0.007760000 0.988319000

F -9.961387000 1.816456000 -0.117310000

C 9.759310000 -0.524232000 -0.046671000

F 9.996961000 -1.403708000 -1.045966000

F 10.582169000 0.526854000 -0.244814000

F 10.139356000 -1.125425000 1.100483000

1. [] Gaussian 09, Revision D.01, Frisch, M. J.; Trucks, G. W.; Schlegel, H. B.; Scuseria, G. E.; Robb, M. A.; Cheeseman, J. R.; Scalmani, G.; Barone, V.; Mennucci, B.; Petersson, G. A.; Nakatsuji, H.; Caricato, M.; Li, X.; Hratchian, H. P.; Izmaylov, A. F.; Bloino, J.; Zheng, G.; Sonnenberg, J. L.; Hada, M.; Ehara, M.; Toyota, K.; Fukuda, R.; Hasegawa, J.; Ishida, M.; Nakajima, T.; Honda, Y.; Kitao, O.; Nakai, H.; Vreven, T.; Montgomery, J. A., Jr.; Peralta, J. E.; Ogliaro, F.; Bearpark, M.; Heyd, J. J.; Brothers, E.; Kudin, K. N.; Staroverov, V. N.; Kobayashi, R.; Normand, J.; Raghavachari, K.; Rendell, A.; Burant, J. C.; Iyengar, S. S.; Tomasi, J.; Cossi, M.; Rega, N.; Millam, J. M.; Klene, M.; Knox, J. E.; Cross, J. B.; Bakken, V.; Adamo, C.; Jaramillo, J.; Gomperts, R.; Stratmann, R. E.; Yazyev, O.; Austin, A. J.; Cammi, R.; Pomelli, C.; Ochterski, J. W.; Martin, R. L.; Morokuma, K.; Zakrzewski, V. G.; Voth, G. A.; Salvador, P.; Dannenberg, J. J.; Dapprich, S.; Daniels, A. D.; Farkas, Ö.; Foresman, J. B.; Ortiz, J. V.; Cioslowski, J.; Fox, D. J. Gaussian, Inc., Wallingford CT, 2009. [↑](#endnote-ref-1)
